# A combination of herbal compound (SPTC) along with exercise or metformin more efficiently alleviated diabetic complications through down-regulation of stress oxidative pathway upon activating Nrf2-Keap1 axis in AGE rich diet-induced type 2 diabetic mice

**DOI:** 10.1186/s12986-021-00543-6

**Published:** 2021-01-19

**Authors:** Golbarg Rahimi, Salime Heydari, Bahareh Rahimi, Navid Abedpoor, Iman Niktab, Zahra Safaeinejad, Maryam Peymani, Farzad Seyed Forootan, Zahra Derakhshan, Mohammad Hossein Nasr Esfahani, Kamran Ghaedi

**Affiliations:** 1 Department of Cell and Molecular Biology and Microbiology, Faculty of Biological Science and Technology, University of Isfahan, Hezar Jerib Avenue, Azadi Sq., Isfahan, 81746-73441 Iran; 2 Department of Medical Biotechnology, Faculty of Allied Medical Science, Iran University of Medical Science, Tehran, Iran; 3 Department of Animal Biotechnology, Cell Science Research Center, Royan Institute for Biotechnology, ACECR, Royan Street, Salman Street, Isfahan, 816513-1378 Iran; 4 Department of Biology, Faculty of Basic Sciences, Shahrekord Branch, Islamic Azad University, Shahrekord, Iran; 5 Legal Medicine Research Center, Legal Medicine Organization, Tehran, Iran; 6 Alzahra Hospital, Isfahan University of Medical Sciences, Isfahan, Iran

**Keywords:** AGE rich diet, Diabetes, Exercise, Herbal drug, Nrf2-keep pathway, Stress oxidative

## Abstract

**Background:**

SPTC is a mix of four herbal components (*Salvia officinalis*, *Panax ginseng*, *Trigonella foenum-graeceum*, and *Cinnamomum zeylanicum*) which might be prevented the development of AGE rich diet-induced diabetic complication and liver injury through activated the nuclear factor erythroid-2-related-factor-2 (Nrf2) pathway. Nrf2, as a master regulator of antioxidant response elements by activating cytoprotective genes expression, is decreased oxidative stress that associated with hyperglycemia and increases insulin sensitivity. the aim of this study was to assess whether the combination therapy of SPTC along with exercise or metformin moderate oxidative stress related liver injurie with more favorable effects in the treatment of AGE rich diet-induced type 2 diabetic mice.

**Methods:**

We induced diabetes in C57BL/6 mice by AGE using a diet supplementation and limitation of physical activity. After 16 weeks of intervention, AGE fed mice were compared to control mice. Diabetic mice were assigned into seven experimental groups (each group; n = 5): diabetic mice, diabetic mice treated with SPTC (130 mg/kg), diabetic mice treated with *Salvia Officinalis* (65 mg/kg), diabetic mice treated with metformin (300 mg/kg), diabetic mice with endurance exercise training, diabetic mice treated with SPTC + metformin (130/300 mg/kg), diabetic mice treated with SPTC + exercise training.

**Results:**

SPTC + exercise and SPTC + metformin reduced diabetic complications like gain weight, water and calorie intake, blood glucose, insulin, and GLUT4 content more efficiently than each treatment. These combinations improved oxidative stress hemostasis by activating the Nrf2 signaling pathway and attenuating keap1 protein more significantly.

**Conclusion:**

Eventually, combined treatment of SPTC with exercise or metformin as a novel approach had more beneficial effects to prevent the development of diabetes and oxidative stress associated with hyperglycemia.

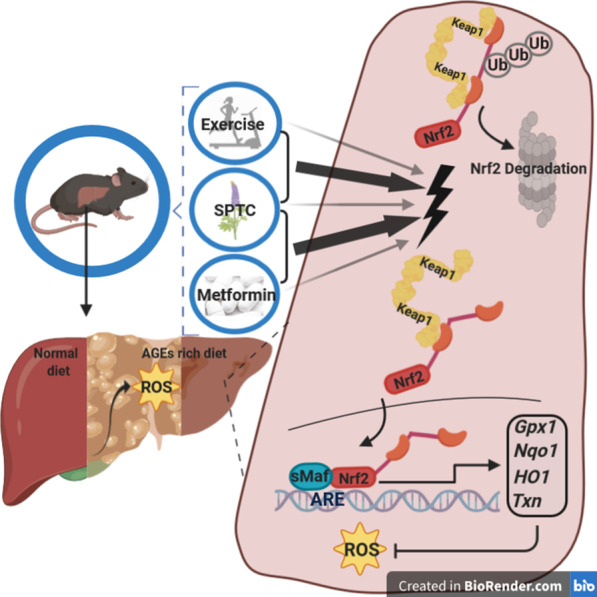

## Introduction

One of the most complex prevalent disorders all over the world is Type 2 diabetes mellitus (T2DM) that is rapidly growing globally. T2DM has been connected with unhealthy dietary habits, including high carbohydrate and advanced glycation end products (AGE) rich meals and sedimentary life-style, and obesity in recent years [1–3]. Heterogeneous pathomolecular cause of diabetes is associated with modulation in several signaling pathways, in which most important of them is stress oxidative. In general, T2DM is characterized by hyperglycemia and hyperinsulinemia that could lead to pro-stress oxidative conditions which could be enhanced levels of reactive oxygen species (ROS), inflammatory factors, and reduction of the antioxidant defenses that cause tissue injury and long term complications of T2DM [4].

The body is equipped with several defense mechanisms against oxidative stress and via this mechanism can return to normal conditions. Nrf2-Keap1 signaling pathway is one of the well-known of these molecular mechanisms. Nrf2 has a cytoprotective role in response to oxidative stress [5, 6] as well as in insulin sensitivity, glucose metabolism, mitochondrial bioenergetics, lipid metabolism, inflammation alleviation, drug metabolism [7]. Nrf2 signaling is triggered by detachment from Keap1 (a protein-rich in cysteine and Nrf2 inhibitor), and translocation of Nrf2 to the nucleus. Subsequently, Nrf2 targets the expression of downstream genes with antioxidant response elements (ARE) in their regulatory regions Such as *Gpx1*, *HO1*, *Nqo1*, and *Txn* [8, 9]. Pathway hierarchy, compounds inducers of ARE related genes expression could independently alter Nrf2-Keap1 structure such as reactive cysteine (Cys) residues in Keap1. Depletion of Nrf2-Keap1 signaling pathway has been closely correlated with T2DM complications like diabetic retinopathy and cardiomyopathy and pancreatic β-cell damage. Actually, Keap1–Nrf2 system activation might be attenuated the damage by stress oxidative and inflammation in diabetes. It was previously reported that Nrf2-inducers could be suppressed stress oxidative markers and subsequently related damage [10].

Nowadays, herbal medicine and physical activity are used as a natural approach to increase antioxidant and anti-inflammatory abilities to combat the micro- and macrovascular complications of diabetes [1]. In traditional oriental medicine, herbal extracts have been extensively used as effective medication against T2DM for a long time [11]. The herbal mixture of SPTC that we used in the present study consists of leaves and seeds aqueous extract of four plants include *Salvia officinalis*, *Panax ginseng*, *Trigonella foenum-graeceum*, and *Cinnamomum zeylanicum* with already identified antioxidant potential. Favorable antioxidant impact of SPTC is due to its flavonoids and polyphenolic components such as ginsenoside, salvianolic acid A, carnosic acid. Intense studies have indicated that treatment with antioxidant reagents might be used as an effective approach in preventing ROS mediated pancreas damage. Indeed, antioxidant therapy could improve secretion of insulin from pancreatic islets and involve in alleviating pancreatic b-cell damage in diabetic individuals [10]. Therefore, SPTC might be accounted for a reduction of T2DM complications through blocking of stress oxidative pathways.

An alternative factor for improving cell metabolic responses and insulin sensitivity is a regular exercise that is prescribed for relieving T2DM. Physical activity is a well-evidenced feasible approach for maintaining metabolic homeostasis and attenuate complications of diabetes [12]. A variety of studies have supported the beneficial effects of exercise, which is mediated through activation of the Nrf2 pathway and increasing endogenous antioxidant defense [13]. Exercise affects through Nrf2 signaling with different mechanisms including increasing ROS lead to oxidation and modification of keap1 cysteine residues [14]. However, moderate exercise-induced ROS could suppress the oxidative damage by increasing adaptive responses of the body and improving antioxidant capacity in tissues. [15]. This adaptive activation have more protective effects when accompanied by antioxidants treatment [16]. Generally, exercise in combination with antioxidants or Nrf2 activator supplementation has more significant effects on oxidative stress situation in the liver [14].

According to evidence, T2DM is an increasing global pandemic and the age of T2DM outbreak continues to decrease [17]. Hence, it is of great significance to explore more efficient therapeutic approach to decrease and delay the development of T2DM condition. One of the first anti-diabetic medication is metformin which has been used as a basic pharmacological therapy in diabetes treatment. However, evidence has proposed that metformin therapy is associated with gastrointestinal tract adverse effects and metformin might be suffered from these side effects. [18]. On the other hand, long term of metformin therapy may lead to prevalence of other diseases like peripheral neuropathy. Accordingly, these aspects highlight a need for herbal drug therapy in diabetic patients. Considering the relation between the stress oxidative, development of diabetes complications, the role of SPTC components, and endurance exercise in improving antioxidant and anti-inflammatory abilities, we hypothesized that utilizing the variance capacity of Nrf2-keap1 complex through exercise and medication with SPTC, might be more efficient to stimulate Nrf2 detachment of keap1 and regulate diabetic related oxidative stress. As both diet type and calorie intake are prominent environmental factors in the pathogenesis and development of T2DM, in the present study we used advanced glycation end products (AGE) rich diet to induce diabetic mice. Since AGE have an important role in liver fibrosis development [20] and liver dysfunction is one of the principal complication of T2DM [21], we aimed to assess to examine aforementioned pathways in the liver tissue. In this study we examine the role of combination therapy with SPTC + exercise and SPTC + metformin compared with each treatment alone to improve T2DM complication and stress oxidative markers include antioxidant capacity and Nrf2-keap1 signaling pathways in AGE rich diet-induced type 2 diabetic mice. Totally, our results showed that SPTC in combination with exercise or metformin lead to decrease T2DM markers and stress oxidative related liver condition.

## Materials and methods

### Ethical issue

All experiments were conducted following the guidelines of the committee of the Royan Institute (IR. ACECR.ROYAN.REC1398.44). Mice were acclimatized to the Royan animal housing condition for two weeks.

### Experimental animal and treatments

Four-week-old male C57BL/6 mice were procured from Royan Institute for Biotechnology (Isfahan, Iran) and were housed in a temperature-controlled facility (24 ± 3 °C and humidity of 65% ± 5), under a 12 h light–dark cycle. They were held ad libitum to access water and foods throughout the study. After adaptation, mice with an approximate weight of 14 ± 2 g, were randomly divided into two groups of control (Ctrl) and diabetic mice (DM) that respectively fed with normal diet and (AGE)-rich diet (Abedpoor et al., Unpublished data) which were obtained from Royan Institute for Biotechnology, for 16 weeks. After ensuring the emergence of DM, the diabetic group was divided into seven groups randomly (n = 5), as follows: (1) diabetic mice (DM group), (2) diabetic mice treated with SPTC (130 mg/kg, DM/SPTC group), (3) diabetic mice treated with *Salvia Officinalis* (65 mg/kg, DM/Sal), (4) diabetic mice treated with metformin (300 mg/kg, DM_/_Met), (5) diabetic mice with endurance exercise training (DM/EX), (6) diabetic mice treated with SPTC + metformin (130 mg/kg + 300 mg/kg respectively, DM/SPTC + Met), (7) diabetic mice treated with SPTC + exercise training (DM/SPTC + EX).

SPTC (*Salvia officinalis* 145 mg, *Panax ginseng* 145 mg, *Trigonella foenum-graeceum* 65 mg, and 25 mg *Cinnamomum zeylanicum*) and *Salvia* were provided as a dried powder. All chemical and herbal drugs were administered as a gavage supplement, five times per week (day 1 up to 5/each week) for 8 weeks.

**Fig. 1:**
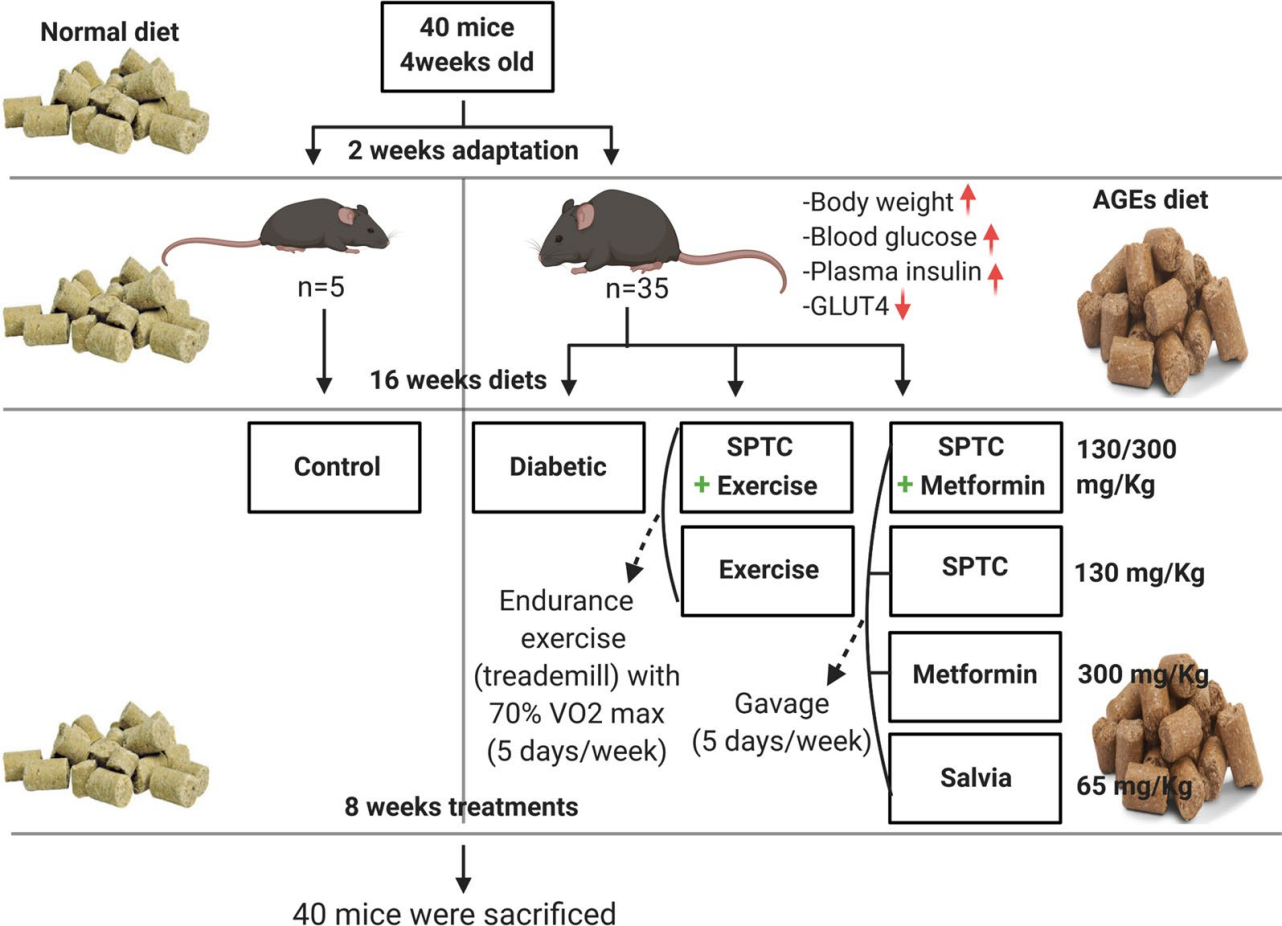
Designed a representation of flowchart. As shown, acclimation was performed for two weeks. mice with an approximate weight of 14 ± 2 g, were randomly divided into two groups of control (Ctrl) and diabetic mice (DM) that respectively fed with normal diet and (AGE)-rich diet for 16 weeks. After ensuring the emergence of DM, the diabetic group was divided into seven groups randomly (n = 5), as follows: 1. Diabetic mice (DM group), 2. Diabetic mice treated with SPTC (130 mg/kg, DM/SPTC group), 3. Diabetic mice treated with Salvia Officinalis (65 mg/kg, DM/Sal), 4. Diabetic mice treated with metformin (300 mg/kg, DM/Met), 5. Diabetic mice with endurance exercise training (DM/EX), 6.Diabetic mice treated with SPTC + metformin (130 mg/kg + 300 mg/kg respectively, DM/SPTC + Met) 7. Diabetic mice treated with SPTC + exercise training (DM/SPTC + EX)

During the experiment period, body weight was measured weekly. In addition, calorie intake, and water drinking were monitored daily. At the end of experiment, mice were sacrificed under xylazine and ketamine anesthesia. Blood and tissue samples were collected for further tests. In the Fig. 1 we designed the flowchart of our experiments.

### Biochemical analyses of plasma glucose, insulin levels, blood ROS, and Total antioxidant capacity

The plasma level of insulin was determined by Ultra-Sensitive Mouse Insulin ELISA Kit (Crystal Chem, US) according to the protocol. Glucose tolerance test (GTT) and fasting blood sugar (FBS) were measured using an Alpha TRAK glucometer and its standard strips (Zoetis, US) as described elsewhere. The blood ROS activity was measured with the OxiSelect Intracellular ROS Assay Kit (Cell Biolabs, USA) according to the manufacturer’s instruction. Total antioxidant capacity of blood was measured by OxiSelect Total Antioxidant Capacity (TAC) Assay Kit (Cell Biolabs, USA).

### Exercise training protocol

Endurance exercise was applied as a type of moderate-high intensity exercise on a motorized treadmill with a 0° incline during 8 weeks (5 days/week). At first, mice were acclimated to treadmill exercise for two weeks, and after that speed and duration of treadmill training were gradually increased at a rate of 3 m/min from 10 to 25 m/min in the final session for 45 min (~ 70% VO_2_ max).

### Quantitative real-time PCR (qRT-PCR)

Total RNA was isolated from the liver using TRIzol reagent (Thermo Scientific, USA). To remove contaminating genomic DNA, samples were treated with DNaseI (TaKaRa, Japan). mRNA was reverse transcribed with 1 μg of total RNA using the cDNA synthesis kit according to the manufacturer’s instruction (TaKaRa). qRT-PCR was performed with CYBR green (TaKaRa, Japan) on an Applied Biosystems real-time PCR thermal cycler (Thermo Fisher Scientific, Waltham, MA, USA). Evaluation of gene expression was carried out according to the 2^−ΔΔct^ method. Accordingly, relative quantification was calculated according to 18 s rRNA as an internal control (Housekeeping). Primers were ordered from micro-gene (Korea), and their sequences are shown in Table 1.

**Table 1:** Primer list

Gene	Forward primer (5′-3′)	Reverse primer (5′–3′)	Annealing temperature (°C)
*Gpx1*	TGAGAAGTGCGAAGTGAATGGTG	TCTCAAAGTTCCAGGCAATGTCG	62
*Txn*	CTCCCCGCAACAGCCAAA	GCAGTCATCCACATCCACTTCAA	62
*Nqo1*	GCCAATCAGCGTTCGGTA	AGTTCATAGCATAGAGGTCAGA	62
*HO1*	GGCTGTGAACTCTGTCTC	ATACCCACCATCACACCCTG	56
*18 s rRNA*	CGGACACGGACAGGATTG	TCGCTCCACCAACTAAGAAC	59

### Western blot analysis

Tissue proteins were extracted using TRI reagent, according to the manufacturer protocol. Proteins were resolved by SDS-PAGE (10%) and transferred to PVDF membranes (Bio-Rad, USA). Membranes were blocked with different blocking buffer containing 10% skim milk (Millipore, USA), and 5% TBST. Then, they were respectively incubated with primary antibodies for 1.5 h (Anti-Nrf2 antibody [1:1000, EP1808Y, Abcam, UK], Anti-Glucose Transporter GLUT4 [1:2000, ab188317, Abcam, UK], anti- β actin antibody (1:500, Sigma, USA), anti-Keap1 antibody [1:1000, ab119403, Abcam, UK] and secondary antibodies (Goat Anti-Rabbit IgG H&L (HRP) (1:20,000, Santa Cruz SC-2301, HRP-conjugated goat antimouse IgG (1:5000, Dako, Japan P0447), for 1 h at room temperature. Blots were detected by an Amersham ECL Advance Western Blotting Detection Kit (GE Healthcare, USA). Image J software (National Institutes of Health, Bethesda, MD, USA) was utilized for quantification of the intensity band.

### Histological studies

Immediately after mice sacrificing, tissues were fixed in 10% buffered formalin and embedded in paraffin. Accordingly, fixed tissues were cut into slices with 5 µm thickness. After deparaffinization and hydration, tissue sections were stained with hematoxylin and eosin (H&E) and finally were observed under light microscopy.

### ROS detection

ROS generation in mice liver was measured by using 2′,7′-Dichlorofluorescin Diacetate (DCFDA) fluorescence method as previously described. Briefly, liver samples (100 mg) were homogenized in 1 mL ice-cooled (4 °C) 40 mM Tris–HCl buffer (pH:7.4). After diluting to 0.25% with the same buffer, the total homogenate of each sample was divided into two equal portions of 2 mL. Approximately 40 μL of 1.25 mM DCFDA was added to one portion, and the same volume of methanol was added to the other portion (Control). After 20 min incubation of all samples in 37 °C, the conversion of DCFH to the fluorescent product DCF was determined at 488 nm excitation and 525 nm emission using BD FACSCalibur Flow Cytometer (Becton Dickinson, USA). The results were evaluated according to DCF fluorescence intensity.

### Total antioxidant capacity

Total antioxidant capacity of tissue and drug samples were determined using Ferric Reducing Antioxidant Power (FRAP) Assay Kit (Naxifer™- TAC Capacity Assay Kit, NS-15012, Iran) as described in manufacturer protocol.

### Statistical analysis

The statistical analyses were carried out using GraphPad Prism 8.5 software (GraphPad Software, San Diego, CA, USA). The paired samples *t*-test was performed to evaluate the diabetic group compared to the control group. Also, One-way analysis of variance (ANOVA) was used to make comparisons between all treatment groups. All experimental results are presented as mean ± SD. *p* value < 0.05 represents significant difference between the samples.

## Results

### Diet enriched with AGE contributed to the development of diabetic complications

**Fig. 2:**
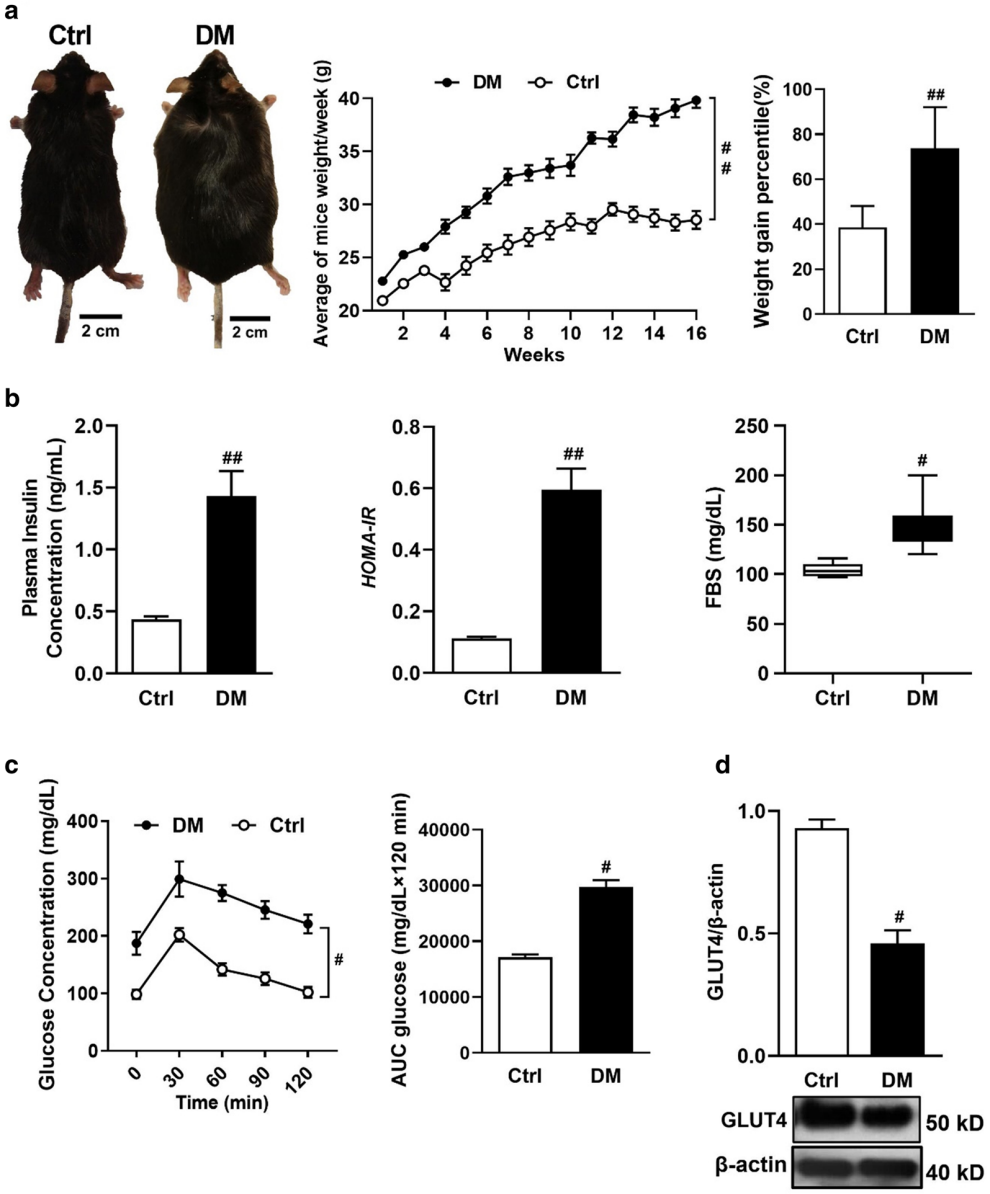
(AGE)-rich diet exacerbates diabetic symptoms in diabetic mice rather than control mice. Control mice (Ctrl) and AGE rich diet-induced diabetic mice, body weight ratios and weight gain percentile (**a**). Measurement of plasma insulin concentration, HOMA-IR and fasting blood sugar (**b**). Glucose concentration and area under the curve glucose (**c**). GLUT4 protein expression in skeletal muscle (**d**). All values were expressed as mean ± SD (n = 5 per group). ^#^*p* < 0.05, ^##^*p* < 0.01 indicate statistically significant difference in the diabetic mice in comparison to the control mice. Data were analyzed by *t*-tests

Our data clearly showed (AGE)-rich diet contributed to increase calorie intake and water drinking of model mice (Table 2). The weight gain percentile of AGE diet group was more than the control group (Fig. 2a). Moreover, the measurement of liver/body weight ratio was shown that AGE diet causes increasing liver/body weight in DM model mice (Table 2).

**Table 2:** Liver/body weight ratio, Calorie intake, and water drinking amount

Groups (n = 5)	Relative liver weight (g liver/g body weight %)	Water consumption (mL/day/mouse)	Calories intake (Kcal/day/mouse)
Control	4.8 ± 0.06	3.65 ± 0.1	3.71 ± 1.45
DM	6 ± 0.22^a^	5.89 ± 0.29^a^	8.9 ± 0.46^a^
DM/Met	4.1 ± 0.12^b^	4.2 ± 0.3^b^	5.38 ± 1.33^b^
DM/SPTC	3.8 ± 0.19^b^	3.85 ± 0.26^b^	6.22 ± 1.2^b^
DM/ SPTC + Met	3 ± 0.3^b^	4.5 ± 0.13^b^	5.23 ± 1.39^b^
DM/Sal	3.5 ± 0.09^b^	4.32 ± 0.15^b^	4.3 ± 1.26^b^
DM/EX	3.9 ± 0.1^b^	4.85 ± 0.6^b^	4.99 ± 1.65^b^
DM/ SPTC + EX	3.1 ± 0.16^b^	3.98 ± 0.16^b^	4.21 ± 1.38^b^

Values are expressed as mean ± SD

^a^Indicates statistically significant difference with control group at *p* < 0.05

^b^Indicates statistically significant difference with DM at *p* < 0.05

**Fig. 3:**
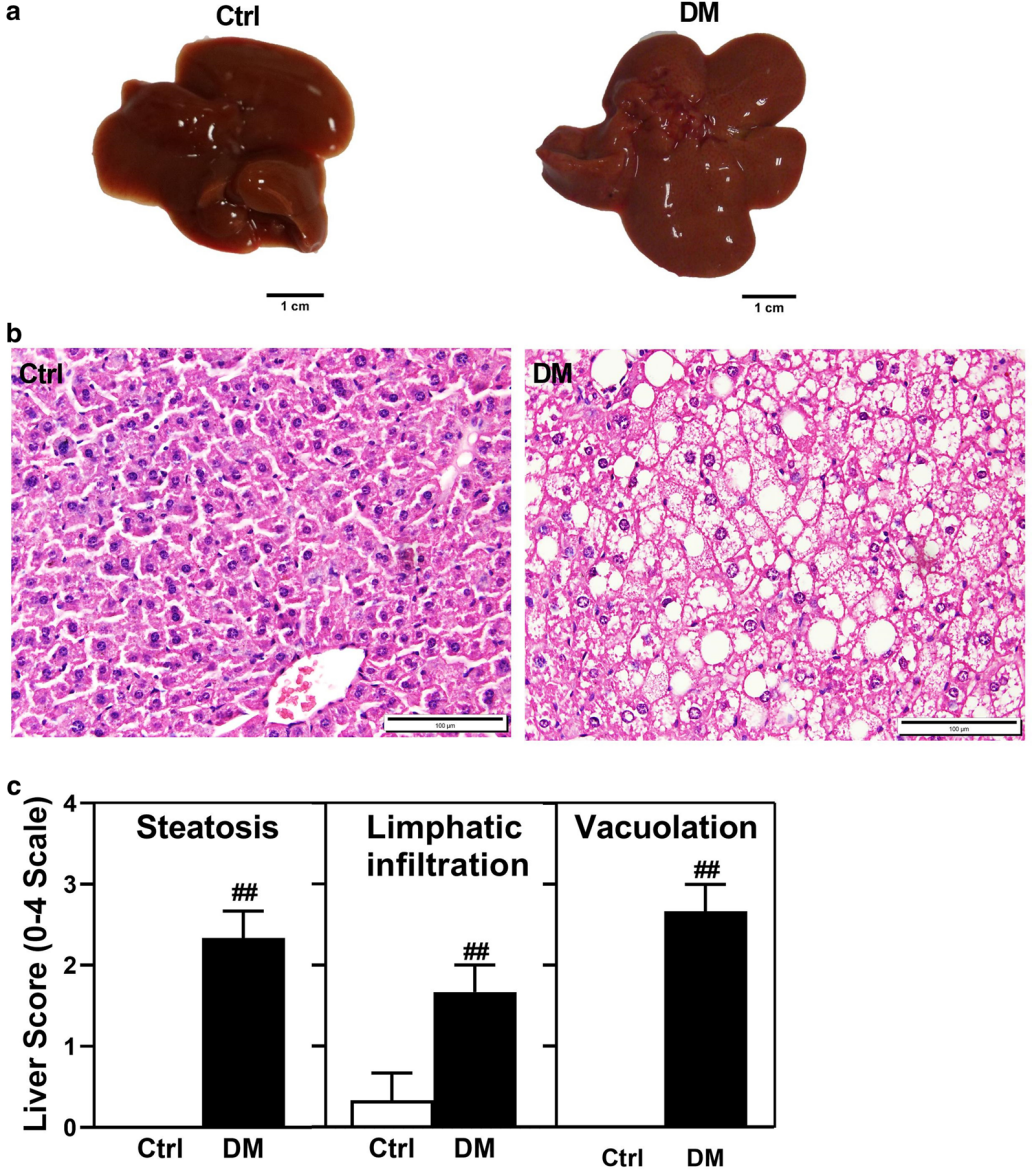
(AGE)-rich diet induces liver damage in diabetic mice rather than control mice. Liver morphology of control and diabetic mice (**a**). H & E staining of liver tissue (**b**). Semi-quantitative evaluation of steatosis, lymphatic infiltration, and vacuolation in liver tissues (**c**). Values were expressed as mean ± SD (n = 5 per group). ^##^*p* < 0.01 indicates a statistically significant difference in diabetic mice in comparison to the control mice. Data were analyzed by *t*-tests. Liver damage were scored by a pathologist after observing the samples of H & E staining

After 16 weeks of (AGE)-rich diet consumption, morphological characteristics of liver tissue are significantly changed (Fig. 3). As a result, (AGE)-rich diet played an essential role in the histopathological characteristics of the liver. HE staining data from diabetic mice demonstrated the development of steatosis, lymphatic infiltration, and vacuolation of the liver (Fig. 3b, c). AGE diet mice also had a larger liver size compare to the normal diet mice (Fig. 3a). Despite such a clear difference in liver characteristics, HE staining of pancreatic and muscle tissue exhibited no vital difference compared with the control group (Data not shown).

### Molecular and biochemical assays

Based on evidence Glucose intolerance and hyperinsulinemia are related onsets for diabetes. Our data indicated that plasma insulin and glucose levels were elevated in mice after 16 weeks by AGE diet. Plasma insulin and glucose tolerance tests (AUC insulin and AUC glucose) were higher in mice with AGE diet compared to mice with a normal diet (Fig. 2b, c). Subsequently, we calculated insulin resistance (HOMA-IR). As depicted, the HOMA-IR index for (AGE)-rich diet mice was significantly amplified (Fig. 2b). Mice on (AGE)-rich diet displayed significantly higher insulin resistance than mice on normal diet.

Insulin resistance triggers Glut4 pathway impairment in skeletal muscle and, finally, glucose uptake deficiency. Determination of GLUT4 expression revealed that this protein level was reduced in mice on (AGE)-rich diet (Fig. 2d).

### (AGE)-rich diet increased oxidative stress in liver

**Fig. 4:**
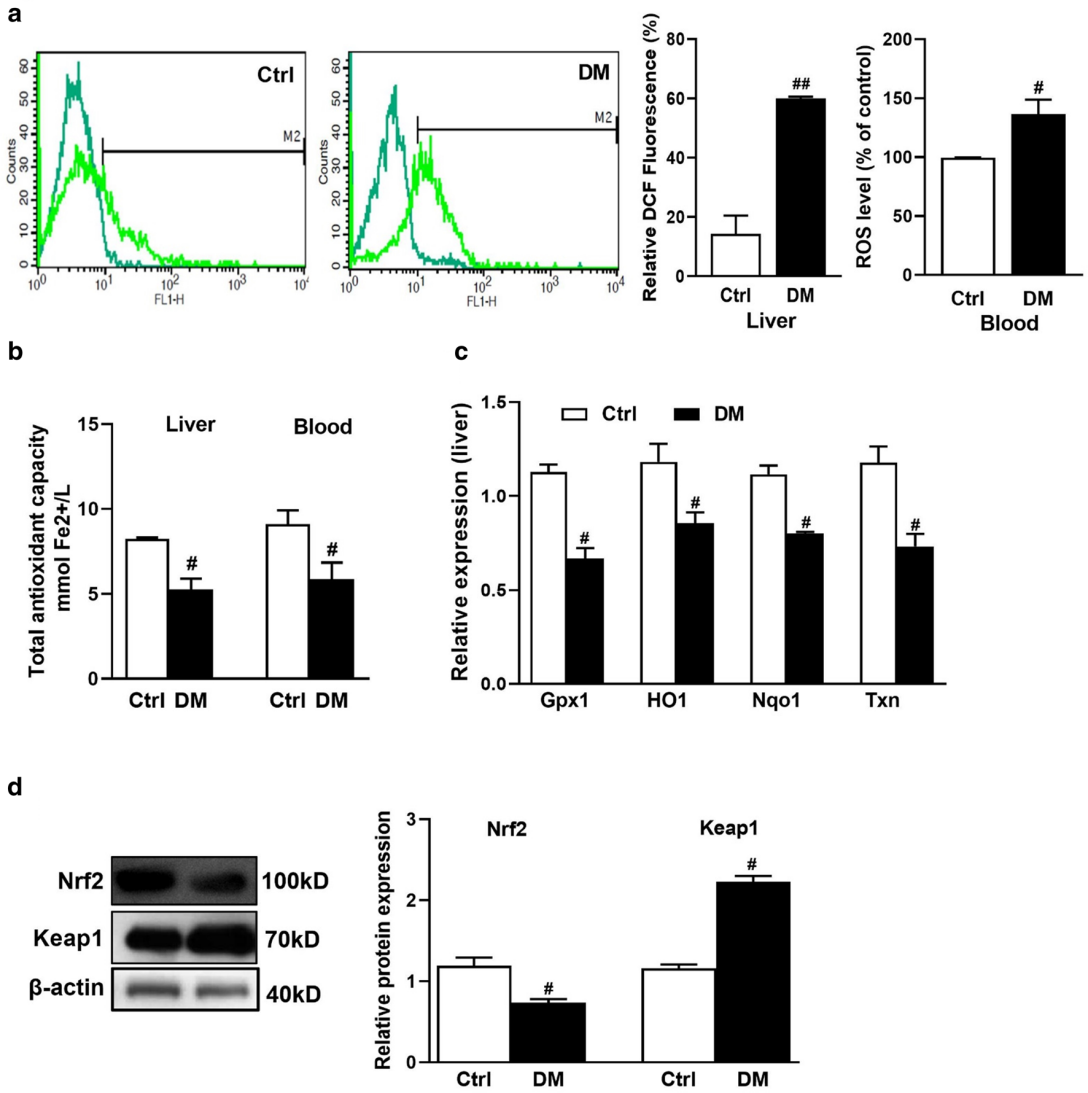
(AGE)-rich diet impaired by the endogenous redox system in liver. Liver ROS production was measured using DCF fluorescence method, blood ROS level was determined by ELISA Kit (**a**). Total antioxidant capacity of liver and blood (**b**). *Gpx1, HO1, Nqo1, and Txn* expression were measured using qPCR and normalized to 18 s rRNA (**c**). Nrf2 and Keap1 proteins expression in liver (**d**). Values were expressed as mean ± SD (n = 5 per group). ^#^*p* < 0.05 indicates a statistically significant difference in diabetic mice in comparison to the control mice. Data were analyzed by *t*-tests

To investigate whether (AGE)-rich diet contributes to making oxidative stress, we analyzed some parameters for oxidative stress in diabetic mice. The data showed that AGE diet raised the amount of ROS in diabetic mice compared to control mice. In the liver, gated cells with DCF (a marker of ROS) increased from 25.03% in the control group to 63.65% in the AGE diet group. As well, AGE diet increased ROS level of blood in diabetic group compared to control (Fig. 4a).

Consistently, the total antioxidant capacity of liver and blood declined in the AGE diet group compared to the control (Fig. 4b). Consequently, we also determined Keap1 and Nrf2 protein levels and related antioxidant genes expression as one of the main signaling pathway involved in oxidative stress regulation. AGE diet resulted in enhancement of the keap1 protein levels whereas, it might be reduced Nrf2 protein levels and downstream antioxidant genes *Gpx1*, *Nqo1*, *HO1,* and *Txn* at the level of transcription in (Fig. 4c, d). (AGE)-rich diet emerged to disturb the balance between ROS generation and antioxidant defense system.

### SPTC, as well as exercise, diminished diabetes complications

After inducing diabetes via (AGE)-rich diet, mice were treated with either SPTC or exercise and a compound type of both interventions. The main body portion of SPTC was *Salvia* extract. Therefore, one group of mice was treated with *Salvia* extract (DM/Salvia). As positive controls, metformin-treated group, and metformin combination with SPTC were also implemented to assess whether SPTC could able to alleviate the diabetes symptoms or not. Despite the reduction of diabetes complications in all groups relative to the control (no received SPTC, metformin or exercise, and *Salvia*, Table 2). Besides, liver/body weight ratio in DM mice reduced partly after 8 weeks of treatment with SPTC, metformin, *Salvia*, and endurance exercise compared to the diabetic group. Furthermore, the simultaneous treatment of SPTC plus exercise and SPTC plus metformin caused a decrease in the ratio of liver/body weight compared to the diabetic group (Table 2).

**Fig. 5:**
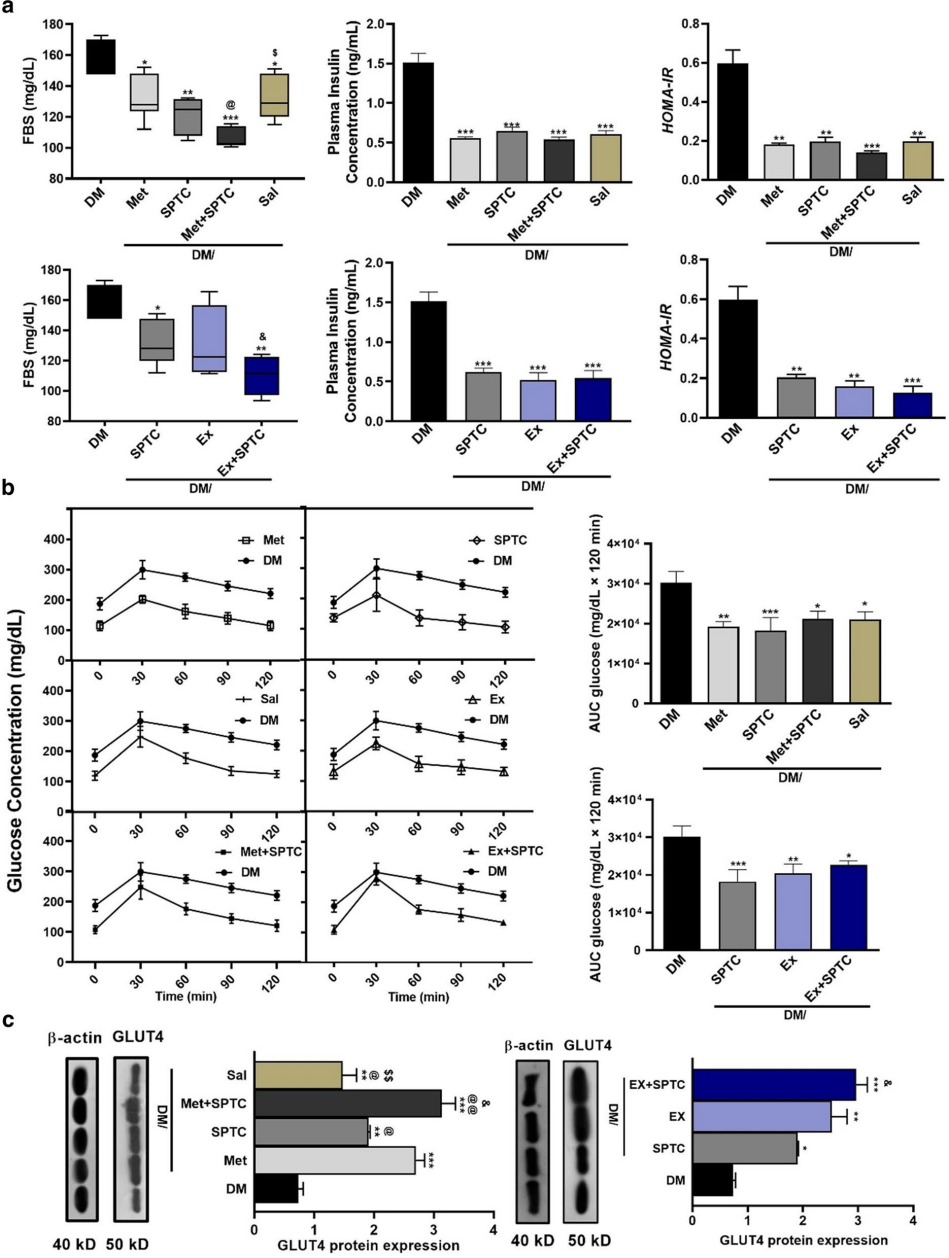
Improvement of diabetic complications of AGE rich diet-induced diabetic mice with different treatment groups. Measurement of plasma insulin concentration, HOMA-IR and fasting blood sugar (**a**). Glucose concentration and area under the curve glucose (**b**). GLUT4 protein expression in skeletal muscle (**c**). All values were expressed as mean ± SD (n = 5 per group). Data were analyzed by one-way analysis of variance (ANOVA) and Tukey's post hoc test

Glucose test tolerance in SPTC, endurance exercise (Ex), and metformin (Met) groups significantly reduced compared with diabetic group (DM). Notably, we found that the SPTC group compared to other groups had more effective and predominately decreased the Blood glucose levels (Fig. 5b).

Moreover, plasma insulin and FBS were significantly decreased after applying all treatment. However, FBS in SPTC plus exercise (SPTC + EX) and SPTC plus metformin (SPTC-Met) groups declined compared to the sole treated group (Fig. 5a). We also calculated HOMA-IR in all groups. It was lower in all treatment groups, and then this reduction was more in combination groups of SPTC plus exercise (SPTC + EX) and SPTC plus metformin (SPTC + Met) than groups of SPTC, metformin (Met), *Salvia* (Sal), and endurance exercise (EX) (Fig. 5a).

The protein expression levels of GLUT4 were measured in skeletal muscle of all treatment groups. The level of GLUT4 was enhanced in all treatment groups compared to the diabetic group. Additionally, in SPTC plus exercise (SPTC + EX) and SPTC plus metformin (SPTC + Met) were significantly increased than SPTC, metformin (Met), *Salvia* (Sal), and endurance exercise (EX) (Fig. 5c).

### Attenuation of oxidative stress was significant in SPTC plus exercise (SPTC + EX), and SPTC plus metformin (SPTC + Met)-treated groups.

**Fig. 6:**
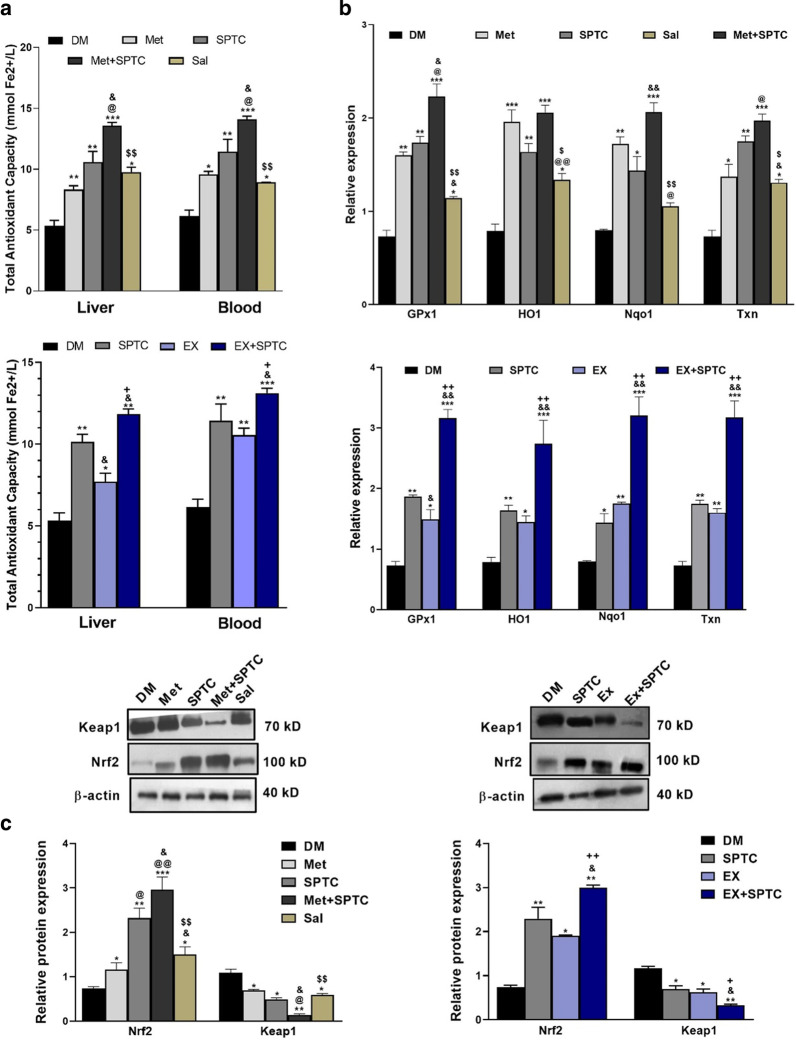
Improvement of oxidative stress condition of AGE rich diet-induced diabetic mice with different treatment groups. Total antioxidant capacity of liver and blood (**a**). *Gpx1, HO1, Nqo1, and Txn* mRNA expression were measured using qPCR and normalized to 18 s rRNA (**b**). Nrf2 and Keap1 proteins expression in liver (**c**). Values were expressed as mean ± SD (n = 5 per group). Data were analyzed by one-way analysis of variance (ANOVA) and Tukey's post hoc test

SPTC, endurance exercise, and metformin treatment amplified the total antioxidant capacity of the liver and blood. Of important antioxidant capacity of SPTC was more than metformin (Met) and it was supposed to be mainly due to the constituents of *Salvia* extract (Additional file 1: Fig. S1). Nevertheless, SPTC plus exercise (SPTC + EX) and SPTC plus metformin (SPTC + Met) restored total antioxidant capacity more than those treatments alone (Fig. 6a). We observed that combinatory treatments increased the abundance of Nrf2 and decreased Keap1 proteins (Fig. 6c). Consistently, SPTC plus exercise (SPTC + EX) and SPTC plus metformin (SPTC + Met) groups indicated a more increased in Nrf2 levels and antioxidant gene expression compared to each treatment alone. Moreover, transcript levels of liver antioxidant genes, including *Gpx1*, *Nqo1*, *HO1,* and *Txn,* were significantly increased in groups of SPTC, exercise (EX), metformin (Met), and *Salvia* (Sal), whereas they were higher in SPTC plus exercise (SPTC + EX) and SPTC plus metformin (SPTC + Met) (Fig. 6b).

## Discussion

The present study indicated more pronounced effects of the combination of SPTC with exercise or metformin on diabetic complications and oxidative stress in diabetic mice which induced with (AGE)-rich diet compared to each treatment alone. Animal model (mouse) was employed in this study to create model of human diabetes mellitus with (AGE)-rich diet and then treated with SPTC plus exercise or metformin. After testing our hypothesis and assisting all aspects of our treatment effects in animal modeling, it might be used in human trials. The primary results of the study were favorable in reducing diabetes complications in mouse model and emerging evidence propose promising therapeutic methods. Further research is needed on this potential approaches. Hence, we could obtain scientifically valid research and expand our study to humans in the future.

In this study, high oxidative stress markers were observed after consumption of (AGE)-rich diet in C57BL/6 mice liver and blood. These results were in good agreement with the previous studies that exhibited the role of AGE and RAGE in the progression of metabolic syndrome like DM by activation of oxidative stress and inflammatory pathways [22, 23]. AGE could be led to ROS production by interacting with its ligand-receptor RAGE and leading to NADPH oxidase activation [24]. It has been explored that in type 2 diabetes, AGE-RAGE signaling inhibited SIRT1 expression and subsequently downregulation of Nrf2 and downstream antioxidant genes [25]. Dysfunction in Nrf2-keap1 signaling as an essential defense system against oxidative stress was indicated in the growing evidence which results in an imbalance between antioxidant and ROS production, and subsequently leads to high blood glucose associated with DM [26]. This is evident that Keap1–Nrf2 signaling pathways notably control insulin secretion and glucose consumption in many tissues and also have a role in regulation of lipid metabolism. Therefore, activation of Keap1–Nrf2 pathways make to maintenance of glucose metabolism and homeostasis. In addition, Nrf2-ARE signaling have a protective role against ROS and inflammation related pancreatic β-cell damage, as a marked pathological impairment in D2M [10].

In the liver, there is a relevant pathway of Nrf2 restricted gluconeogenesis-related gene expression which plays a role in insulin sensitivity, maintenance of normal level of blood glucose and obesity prevention [27]. Moreover, some reports claimed that treatment with Nrf2 inducers like SFN lead to inhibition of the hepatic deterioration through Nrf2/ARE signaling pathways activation in some liver disease [10]. In the present manuscript we have tried to figure out an interconnection with activation of Nrf2-keap1 signaling pathway as a multifactorial process and modulation of oxidative stress associates with diabetes.

Additionally, we investigated significant depletion of blood glucose and insulin levels, insulin resistance, and GLUT4 protein level was increased in after treatment with SPTC, endurance exercise training, metformin, and combination treatment of SPTC plus exercise (SPTC + EX) and SPTC plus metformin (SPTC + Met). In general, diabetic complications decreased further in combination treatment of SPTC plus exercise (SPTC + EX) and SPTC plus metformin (SPTC + Met) compared to each treatment of SPTC, exercise (EX), *Salvia* (Sal), and metformin (Met). Moreover, we found that SPTC and exercise (EX) might be had strong antioxidant effects on diabetic mice which fed (AGE)-rich diet as observed by elevation of Nrf2 protein level and *HO1, Gpx1, Txn*, and *Nqo1* genes expression and depletion of Keap1 protein level in liver. Furthermore, combination treatment could be had the highest additive effect in the induction of antioxidant capacity of the liver and blood rather than each treatment alone.

Antioxidant therapy as new clinical trials is promisingly used for medicinal purposes [28]. Currently, phenolic compounds like flavonoids and polyphenols which have natural origins and lesser side effects compared to synthetic drugs are widely suggested to treat diabetic damage [29]. Given that, mixed therapy of antioxidant herb with other antidiabetic applications had more combination effects, we hypothesized that Nrf2 signaling antioxidant activator might be affected with exercise and metformin than each treatment alone. Our results showed that combination treatment of SPTC plus exercise and SPTC plus metformin exerts more capable effects in diabetic injury and modulation of oxidative stress. Also, our findings indicated that these combined interventions in this study strongly caused more control oxidative stress by activation of the Nrf2-keap1 signaling pathway. This is possibly mediated by various complementary mechanisms that lead to Nrf2 activation and thereby induction of related downstream cytoprotective gene expression.

*Salvia officinalis* with significant bioactive composition have been extensively used for the treatment of type 2 diabetes in herbal medicine [30]. Carnosic acid is one of the main polyphenolic diterpenes of *Salvia officinalis* and was found as an activator for Nrf2-keap1 transcriptional pathway. Takumi and colleagues have observed that Carnosic acid led to phase 2 enzymes induction by binding to keap1 cysteines and Nrf2 nuclear translocation [31]. Salvianolic acid A is another robust antioxidant compounds found in herb *Salvia officinalis* and was reported to activate Akt/mTORC1 signaling which results in Nrf2 phosphorylation [32, 33]. Ginseng, as a traditional anti-diabetic herb was demonstrated to inhibit pro-inflammatory factors and promotes the Nrf2/keap1 pathway. Ginsenoside Rg1, the main active ingredient of Ginseng, by decreasing NLRP3 leads to down-regulation of ILb, which further restricted NF-κB activation [34]. NF-κB unidirectionally acts as an antagonizing factor against the Nrf2 through depletion of MAF protein, which interacts with Nrf2 [35].

As we reported, exercise training and metformin treatment diminished diabetic complications and induced Nrf2-keap1 signaling pathway. Nonetheless, as mentioned, their effects were more pronounced in combination with SPTC. It is presumable that different mechanisms of each treatment had possibly additive effects to improve DM and reduced oxidative stress injury. Metformin, as a first-line antidiabetic medicine, was reported to induce Nrf2 gene expression along with BACH1 protein reduction [36]. BACH1 protein was recognized as an Nrf2 repressor by binding to Nrf2 sites and causes inhibition of antioxidant response element genes expression [37].

Exercise with increasing ROS moderately and oxidation of cysteines residues of keap1, leads to the formation of disulfide bridges, alteration of the Nrf2/Keap1/Cul3 Complex structure and Nrf2 detachment from keap1 [13, 14]. In response to inducing ROS formation during physical training, Nrf2 is activated through oxidation of the cysteine residues in Keap1 and subsequently Nrf2 dissociation [38]. Moreover, it has been reported that PGC-1α modulates the systems to tolerate induced ROS production in exercise. Exercise could be induced PGC-1α protein expression and activation. Therefore, PGC-1α might interact with Nrf2 and causes antioxidant gene expression. This interaction and induction of Nrf2 related pathways are involved in different adaptations to long term training [39].

Besides, our finding showed that the antidiabetic approach combining the protective effects of SPTC is association with exercise training and metformin and have more effective in modulating oxidative stress associated with type 2 diabetes. This study generally reported that SPTC plus exercise and SPTC plus metformin dramatically reversed AGE induced diabetic changes and decreased oxidative stress injury more than each treatment alone. Moreover, all treatments of SPTC, exercise, metformin, *Salvia* and their combinations reduced blood glucose and insulin levels, insulin resistance and increased GLUT4 protein level. Moreover, all treatment amplified Nrf2 protein levels and its antioxidant gene expression of *HO1, Nqo1, Gpx, and Txn* and declined Keap1 protein level in diabetic mice liver. These favorable effects were more remarkable in combination treatment. Consequently, we suggested that combination therapy of SPTC plus exercise (SPTC + EX) or metformin (SPTC + Met) could serve as a potential intervention approach through activating the Nrf2-keap1 pathway and alleviating oxidative stress associated with diabetes mellitus. We hope that future studies will obtain further experimental data to support our results.

## Conclusion

Our data demonstrated beneficial effects of SPTC supplementation in combination with exercise (SPTC + EX) or metformin (SPTC + Met) in (diabetic mice than each treatment alone, suggesting that SPTC + EX and SPTC + Met could be an accessible and therapeutic approach related to lifestyle in diabetic individuals.

## Supplementary Information

**Additional file 1: Fig. S1**. Comparative antioxidant capacity of SPTC, Salvia against metformin. As indicated Salvia extract had significant inducing effect on antioxidant capacity of the live and blood in DM mice.

## Data Availability

All of the raw data and the rest of the materials are remained in Royan Institute for Biotechnology and are available upon request.

## Abbreviations

AGE: Advanced glycation end products
ANOVA: One-way analysis of variance
ARE: Antioxidant response elements
Cys: Cysteine
DCFDA: 2′,7′-Dichlorofluorescin diacetate
T2DM: Type 2 diabetes mellitus
FBS: Fasting blood sugar
FRAP: Ferric reducing antioxidant power
Gapdh: Glyceraldehyde-3-phosphate dehydrogenase
GTT: Glucose tolerance test
H&E: Hematoxylin and eosin
qRT-PCR: Quantitative real-time PCR
ROS: Reactive oxygen species
