# Correction to: A combination of herbal compound (SPTC) along with exercise or metformin more efficiently alleviated diabetic complications through down-regulation of stress oxidative pathway upon activating Nrf2-Keap1 axis in AGE rich diet-induced type 2 diabetic mice

**DOI:** 10.1186/s12986-021-00550-7

**Published:** 2021-03-23

**Authors:** Golbarg Rahimi, Salime Heydari, Bahareh Rahimi, Navid Abedpoor, Iman Niktab, Zahra Safaeinejad, Maryam Peymani, Farzad Seyed Forootan, Zahra Derakhshan, Mohammad Hossein Nasr Esfahani, Kamran Ghaedi

**Affiliations:** 1 Department of Cell and Molecular Biology and Microbiology, Faculty of Biological Science and Technology, University of Isfahan, Hezar Jerib Avenue, Azadi Sq., Isfahan, 81746-73441 Iran; 2 Department of Medical Biotechnology, Faculty of Allied Medical Science, Iran University of Medical Science, Tehran, Iran; 3 Department of Animal Biotechnology, Cell Science Research Center, Royan Institute for Biotechnology, ACECR, Royan Street, Salman Street, Isfahan, 816513-1378 Iran; 4 Department of Biology, Faculty of Basic Sciences, Shahrekord Branch, Islamic Azad University, Shahrekord, Iran; 5 Legal Medicine Research Center, Legal Medicine Organization, Tehran, Iran; 6 Alzahra Hospital, Isfahan University of Medical Sciences, Isfahan, Iran

## Correction to: Nutr Metab (Lond) (2021) 18:14 10.1186/s12986-021-00543-6

Following the publication of the original article [1], the authors identified an error in the funding note.

Incorrect funding note:

A part of this research was funded partially by NIMAD (National Institute for Medical Research Development) [no. 971130].

Correct funding note:

This research was funded by NIMAD (National Institute for Medical Research Development), Project no. 971130.

The author group has been updated above and the original article [1] has been corrected.
